# Dendritic Cells in Cord Blood Transplantation: A Review

**DOI:** 10.4061/2011/539896

**Published:** 2011-06-16

**Authors:** Marta Isabel Pereira, Artur Paiva

**Affiliations:** ^1^Clinical Hematology Department, Coimbra University Hospitals, Coimbra, Portugal; ^2^Histocompatibility Center of Coimbra, 3000-075 Coimbra, Portugal

## Abstract

Dendritic cells (DCs) are a heterogeneous population of antigen-presenting cells derived from hematopoietic progenitors that bridge the transition between the innate and adaptive immune responses, while maintaining self-tolerance and Th1/Th2 homeostasis, by priming other cells in either an immunogenic or tolerogenic direction. Through their role in both innate and adaptive immunity, DCs play a major part in transplant engraftment and rejection and in graft-versus-host disease (GvHD). Preferentially tolerogenic or immunogenic DC subtypes offer targets for immunotherapy, to optimize transplant success rates and prolong disease-free and overall survival. Cord blood DCs are immature and preferentially tolerogenic, due to maternal-fetal tolerance, leading to better graft acceptance and immune reconstitution and explaining the lower incidence and severity of GvHD in CB transplantation, despite donor-host mismatching. Manipulation of DC maturation and cell loading with tumor-antigens can direct antitumor immunity and target minimal residual disease, as demonstrated for acute myeloid leukemia, optimizing the graft-versus-leukemia effect.

## 1. Dendritic Cells

Dendritic cells (DCs) are a heterogeneous population of potent lineage-negative HLA-DR^+^ antigen-presenting cells (Apcs), derived from CD34^+^ hematopoietic progenitors that are present in small numbers in solid tissues and peripheral blood, and that bridge the transition between the innate immune response and adaptive responses, through their activation of CD4^+^ T-cells, CD8^+^ T-cells, and B-cells, while maintaining self-tolerance. 

Recent data suggest that DCs arise from multilymphoid progenitors, along with lymphoid cells, monocytes, and macrophages [1], contradicting the classic and widely accepted model of early lymphoid-myeloid lineage segregation between the two main DC subsets, which can be differentiated through the expression of the inactivated-C3b receptor 4 (complement transmembrane protein) integrin alpha X chain (ITGAX or CD11c). This model postulates that CD11c^−^ CD123^high^ CD33^−^ CD16^−^ plasmacytoid dendritic cells (pDc) derive from a lymphoid progenitor cell, whereas CD11c^+^ CD123^dim^ CD33^+^ CD16^−^ myeloid dendritic cells (mDc) originate in a myeloid precursor cell [2–4]; the mDc subset can be further divided into distinct subpopulations, according to the expression of blood dendritic cell antigens—BDCA1^+^ (CD1c^+^) DC and BDCA3^+^ (CD141^+^) DC. The remaining Bdcas, BDCA2, and BDCA4 are expressed by pDc [4]. A third subtype of DC with a distinct immunophenotype has been described, comprising monocytoid-related CD16^+^ CD14^−/low^ CD123^inter^ CD33^inter^ DC, with a possible origin in differentiated mature monocytes which have downregulated or lost the monocyte marker CD14 [5–7].

Myeloid dendritic cells (also called DC 1) express toll-like receptor (Tlr) 2 and Tlr 4 and secrete Il-12, which alters the Th-cell balance in a Th1 direction, while pDc (also known as DC 2), physiologically residing in primary and secondary lymphoid organs, express Tlr 7 and Tlr 9 and secrete type I interferons (Ifn) and prime a Th2 response. The latter subtype, through its ability to differentiate *in vitro* into mature classic DC, would provide the link between the innate response and the adaptive one [8]. The balance between Th1- and Th2-type immunity is thus dependent on the equilibrium between the different subsets of DC, the loss of which could lead to immune dysregulation, as suggested by observations of an epidemiological inverse relationship between infancy/childhood infections and an atopic potential, the basis of the so-called “hygiene hypothesis” [9], which is still controversial.

The pathogenic role of DC dysfunction (and its role as a potential therapeutic target) is the focus of continuing research, with descriptions of DC involvement in pathologic changes which have been associated with Th1/Th2 dysregulation, such as autoimmune disease (including rheumatic, neurologic and endocrine diseases [10–12], characterized by a Th1 response [13]) and allergy (including asthma, atopic dermatitis, and drug hypersensitivity [14–16], with a skew towards Th2 [13, 17]), and pDc being implicated in diseases with a Type I Ifn-signature [18]. The rebalancing of Th1- and Th2-type responses, through Th2 stimulation in autoimmunity and Th1 shift in allergy, could be harnessed therapeutically [9, 19, 20]. Han et al. highlighted the role of DC in the re-regulation of the Th2 shift in allergy, by showing that DC isolated from *Chlamydia muridarum*-infected mice could inhibit allergen-induced systemic and local eosinophilia, on adoptive transfer [20].

The applications of immunotherapeutic DC vaccination, as a way of enhancing host anticancer immunity, is another developing field; the administration of DC loaded with leukemic cell antigens is a promising experimental treatment for minimal residual disease in acute myeloid leukemia and was the focus of a recent review by Van Den Ancker et al. [21].

Antigen presentation by DC can direct immune responses in both an immunogenic (stimulatory) and a tolerogenic direction [22], with various stages of maturation being associated with opposing functions. In a steady state, DC help to maintain self-tolerance, which is a central issue in immune homeostasis; in tissues, challenge by pathogens with stimulation of pattern-recognition receptors induces the maturation of immature sentinel resident DC, which then migrate towards the draining lymph nodes to present antigens to T cells and prime an adaptive specific response. However, the traditional view of a clear segregation between immature/tolerogenic and mature/immunogenic capabilities seems not to be a correct representation of DC function [23]; the induction of tolerance has been reported in immature, and partially mature DC phenotypes (similar to steady-state migratory veiled DC which tolerize lymph node T-cells towards self), whereas only the fully mature stage of DC differentiation would be immunogenic and able to release proinflammatory cytokines [24], with inflammatory stimuli converting a tolerogenic signal to a stimulatory one [22]. Current knowledge suggests that the induction of regulatory T cells (T_reg_), and not just the lack of inflammatory signals, contributes to the maintenance of tolerance by immature DCs [3, 23]. 

Various authors have shown that DC maturity/immaturity can be manipulated, further optimizing the immunotherapeutic potential of these cell populations. DC maturation can be stimulated *in vitro* with increasing concentrations of growth hormone, resulting in increased Il-12 secretion and increased lymphocyte activation [25], with a Th1 shift, while the early addition of tumour-necrosis factor alpha (Tnf-*α*) to umbilical cord blood (cord blood, Cb) mononuclear cell cultures enhances cell survival and increases DC maturation markers (CD80, CD83, CD86, and Hla-Dr), also heightening Il-12 secretion by mature DC [26].

## 2. Dendritic Cells in Transplantation

One of the main complications of solid organ transplantation is the rejection of the engrafted tissue by the host's immune system, with loss of function. In allogeneic hematopoietic stem cell or bone marrow transplantation (allo-Bmt), the opposite can also happen, with the engrafted tissue rejecting the host's immune system, a phenomenon known as graft-versus-host disease (GvHD), which is responsible for a significant fraction of morbidity and both early [27] and late post-transplant death [28], due to organ damage and infection, in a context of immunodeficiency. A timeline has been used to separate acute GvHD (aGvHD), which takes place up to 100 days post-transplant and typically involves skin, the gastrointestinal tract, and the liver, from chronic (cGvHD) starting from the 100th day onward; cGvHD presents with a generalized systemic involvement and shares many of its features with autoimmune diseases, suggesting different pathogenic events from aGvHD [29]. The importance of DC and the Th1/Th2 homeostasis in autoimmunity has been described above. 

The engrafted immune system, however, can also mount a response against the host's leukemic clone, a beneficial immunotherapeutic effect of allo-Bmt described as the graft-versus-leukemia (GvL) effect, which can reduce primary disease relapse rates [30] but is diminished by the effort to combat GvHD, since part of the GvL effect is proportionally related to the intensity of GvHD [31, 32]. The benefit of the decrease in relapse rates due to GvL can be diluted by the increase in early and late death caused by GvHD, resulting in no effect on overall survival. As there also appears to be a direct antileukemic effect which is independent of GvHD [32], stem-cell transplantation should ideally evolve to exploit this effect while diminishing the impact of GvHD, to improve disease-free survival and overall survival.

Alloreactive T cells are responsible for the rejection of allografts through major histocompatibility complex (Mhc) cross-recognition. GvHD is due to donor (graft) T-cell recognition of Mhc and minor histocompatibility antigen (miHAg) mismatches between graft and host, with aGvHD depending on donor CD8^+^ T-cells and cGvHD originating with donor CD4^+^ T-cells [33, 34]. 

The *in vitro* and *ex vivo* manipulation of the stage of DC maturation, with a selection of the tolerogenic preferentially immature stage, has previously been shown to be able to induce allotolerance by specifically targeting alloreactive T-cells, suggesting a role for the use of manipulated donor or recipient DC in the management of transplant engraftment and rejection [35]. Animal models of solid organ transplantation have shed light on the importance of DC in alloimmunity; in rats, the transplantation of a donor heart deprived of its DC population and repopulated with host-DC prior to transplantation, in an effort to favour tolerance, found, instead, an acute rejection of the organ, starting *in situ* in the donor [36], demonstrating that both host and donor immune cells play an important role in GvHD. Several published studies have further clarified the role of donor and host Apcs. In the murine model, it has been shown that, immediately after transplantation, and before clearance of host DC, the latter activate donor CD8^+^ T cells which, in turn, start the aGvHD response [33, 34, 37]. cGvHD, on the other hand, appears to be dependent on either host or graft Apcs [33, 34]. In humans, the role of DC in transplantation is perhaps best exemplified by the rapid acute rejection of skin allografts, which has been attributed to the skin's richness in a resident skin-homing DC population (Langerhans' cells) [38], and which has limited the field of skin transplantation. The early finding that the immunosuppressive anti-GvHD drug sirolimus (rapamycin) exerts its effect through DC modulation (including modulation of maturation [39], macropinocytosis and endocytosis [40], antigen uptake [41], and signaling and apoptosis [42–44]) further emphasized the role of Dc in the GvHD response. Klangsinsirikul et al. have supported the previous findings by showing that the elimination of the host Apcs diminishes the intensity of GvHD [45], while Sato and colleagues have shown that host-derived regulatory DC (Dc_reg_) generated *in vitro* that express Mhc Class II and lack expression of immune costimulatory molecules were more effective in preventing GvHD than classic tolerogenic DC, through the induction of T-cell anergy due to diminished co-stimulation, despite the presence of a potent antigenic signal [46, 47]. The same authors identified a naturally occurring population of Dc_reg_ (CD49b^+^ CD200R3^+^) in a murine model of Mhc-compatible, miHAg-incompatible allo-Bmt, which suppressed cutaneous cGvHD through a decrease in proinflammatory cytokines and a diminished donor CD4^+^ T-cell alloreaction [48]. Their work in a murine model has also shown that Dc_reg_ cells are associated with a decreased rate of post-transplant leukemic relapse, demonstrating the possibility of obtaining a strong GvL effect separate from the GvHD response [46] and further highlighting the importance of the manipulation of DC subpopulations as a way to increase disease-free survival in progenitor cell transplantation. In fact, recent studies [49, 50] have identified a population of CD8*α* ^+^ T-cell receptor (TCR)^−^ facilitating cells (FCs) that enhance allo-Bmt engraftment and tolerance, and decrease GvHD, and have characterized the main subpopulation of FC as plasmacytoid precursor dendritic cells (p-preDCs) which can induce antigen-specific T_reg_ .

Tolerogenic DC, through their ability to induce T_reg_ expansion, have also been shown to confer protection from autoimmune diseases. Il-10, transforming growth factor beta (Tgf-*β*), granulocyte colony-stimulating factor (G-Csf), hepatocyte growth factor (Hgf), and vasoactive intestinal peptide (Vip) have all been found to modulate DC maturation, favoring the differentiation of tolerogenic DC, an ability which may be harnessed therapeutically for the treatment or prevention of autoimmunity, graft rejection and GvHD [23].

## 3. Dendritic Cells in Cord Blood Transplantation

Immunologically, umbilical Cb differs from adult peripheral blood, as a consequence of its function, simultaneously reflecting the need to prevent mother/fetus alloimmunization and the diminished immune stimulation due to the reduced antigenic load of the intrauterine environment. 

Among the differences described are a diminished percentage of DC and specific CD14^+^ monocyte subsets [51]; nevertheless, Cb is a rich source of hematopoietic stem cells and progenitor cells, and immune cells (including DC) at an immature stage of differentiation. In fact, the phenotype of fetal and neonatal/infant DC (as determined by flow cytometry) is skewed towards immaturity, when compared with adult DC, with a suggestion of decreased ability to take up antigens through IgG-mediated mechanisms (as revealed by a decreased expression of the IgG receptors CD32 and CD64) and reduced co-stimulation [52]. A decrease in co-stimulation was one of the characteristics identified by Sato et al., in Dc_reg_, in the studies described above [46–48]. Furthermore, other authors have found that Cb DCs secrete less Tnf-*α* and Ifn-*α*, after stimulation, than Pb DCs [51]. Plasmacytoid and mDc responses to Tlr 4 agonists (bacterial lipopolysaccharide) and Tlr 9 agonists (CpG oligonucleotides) are decreased in the neonate and infant, when compared to adult responses and increase during the first year of life [53]. Other studies, however, have suggested that Cb Dc have better antigen-presenting capabilities than peripheral blood or bone marrow DC, as represented by an increase in antigen-positive endosomes on fluorescent microscopy [54], findings which can also be viewed in light of Sato's description of potent antigen signaling without co-stimulation [46–48]. 

The overall status of the fetal/neonatal immune system seems to be skewed in a Th2 direction, with a decrease in adaptive responses, which is in part responsible for a heightened susceptibility to infections in infancy [55, 56] (and consequent dependence on the mother's immune system), but which is necessary to avoid Th1-dependent mother/fetus alloimmunization [57]; the mature Th1/Th2 balance is acquired by the naïve immune system through exposure to microbial antigens, a process which is necessary to avoid immune dysfunction such as allergy or autoimmunity [58], as previously described.

The diminished tendency for alloimmunization which is characteristic of Cb could theoretically be harnessed for transplantation, with reduced alloreactivity between host and donor. Consequently, human Cb was used for the first time as the source of hematopoietic stem cells for transplantation just over two decades ago by Gluckman et al. [59] and, since that first description, Cb transplantation has been observed to be associated with less frequent and less severe GvHD than allo-Bmt [60], allowing for a higher degree of mismatching between donor and host.

Several factors have been identified which contribute to the decreased incidence of GvHD with Cb, involving both adaptive and innate immunity, and including phenotypic or functional immaturity, reduced proinflammatory cytokine-producing T-cell populations and increased immunosuppressive factors [61–65]. That the diminished GvHD response could also in part be due to the differences in the DC populations found in the two tissues, is demonstrated by the different expression of the human leukocyte antigen (Hla)-G [66] and the lower expression of cell-surface markers involved in DC/T-cell interaction [67]. Cb monocytes and Cb CD16^+^ CD14^−/low^ DC produce lower basal levels of cytokines (Il-1*β*, Il-6, Il-12, and Tnf-*α*), when compared to peripheral blood, with identical responses to stimulation, reflecting the reduced antigen stimulation *in utero*, but suggesting that the ability to respond to stimulation shown by monocytes and CD16^+^ CD14^−/low^ DCs reflects cellular maturity [5]. The analysis of the three DC subpopulations has pointed at lower absolute and relative numbers of CD16^+^ CD123^inter^ CD33^inter^ DC in Cb [68], the subset that expresses the highest amounts of proinflammatory cytokines [69, 70], which would contribute to the relative immune immaturity of Cb [68].

Several authors have found a decrease in the frequency of monocytes expressing Il-12 and Tnf-*α* in Cb [5, 51], which could help to explain the decreased Th1 response and alloimmunization described for fetal/neonate blood and, consequently, the reduced inflammation and diminished GvHD incidence and severity [5] that has been reported in Cb transplantation. On the other hand, the factors listed above can also explain the increased incidence of infectious complications which has been described by some authors in Cb transplantation [71], a parallel of neonate/infant infectious susceptibility. The complex relationship between infectious complications and GvHD was clinically demonstrated by initial unexpected descriptions of reduced cytomegalovirus (Cmv) reactivation rates in patients undergoing sirolimus prophylaxis of GvHD after allo-Bmt, which led Marty et al. to publish a retrospective analysis that confirmed a protective effect against Cmv [72], a result which could take into account both the role of Cmv infection on DC function impairment [73], and the modulatory effects of sirolimus on DC function, described above.

Adding to the putative causes of the higher Cb transplantation success, is the increase in CXCR4 in Cb attributed to immune immaturity, and which could lead to improved engraftment [5] and decreased graft rejection, with a potential for improving disease-free survival. The described ability of Cb DCs to efficiently induce T_reg_ expansion, despite other markers of immaturity, contributes to graft tolerance in Cb allo-Bmt [51].

Immune reconstitution after transplantation has been compared for Cb and allo-Bmt; Moretta et al. found that reconstitution was not only comparable between the two progenitor cell sources, but Cb recipients actually showed a higher number of B cells, with a better CD4^+^ T-cell recovery in unrelated Cb recipients, which the authors attributed to the decreased GvHD effect [71]. In human allo-Bmt, Arpinati et al. suggested that DC reconstitution was impaired by the presence of an aGvHD response, as well as by steroid treatment [74], which would point at a bidirectional influence, with DC regulating GvHD, and the latter impacting on DC reconstitution. This bidirectional effect of GvHD on the success of regaining a normal population of DC could also partly justify the very high incidence of infectious complications described in GvHD [75]. Thus, Cb transplantation could help to reduce GvHD-related infectious complications, though the positive benefit might be offset by the infectious susceptibility described above.

Finally, Cb GvL effect has also been favourably compared to peripheral blood, with recent studies showing that Cb DC enhance the proliferative capability and antileukemia effect of Cb cytokine-induced killer (Cik) cells, when compared to Cb Cik cells alone, with a higher proliferative ability than peripheral blood DC-Cik cells [76]. 

In conclusion, DC populations, through their central role in both innate and adaptive immunity, play a major part in transplant acceptance, engraftment, and rejection, and in graft-versus-host disease, through either their tolerogenic or immunogenic functions, and the maintenance of the Th1/Th2 balance. The identification of population subtypes with a preference for tolerance or for immune stimulation will offer targets for immunotherapy and cellular manipulation, to optimize transplant success rates, decreasing early and late transplant-associated death, as well as primary disease relapse, prolonging disease-free survival and overall survival. 

Cord blood DC are immature, as a consequence of necessary maternal-fetal tolerance and fetal immune naïveté, which renders them preferentially tolerogenic and, therefore, theoretically associated with better graft acceptance and immune reconstitution, while helping to explain the lower incidence and severity of GvHD in Cb transplantation, even in the presence of donor-host mismatching. 

The* ex vivo* or *in vitro* manipulation of DC, through the induction of maturation and cell loading with specific tumor antigens, can direct antitumor immunity and target minimal residual disease, as demonstrated for acute myeloid leukemia, optimizing the graft-versus-leukemia effect and dissociating it from the GvHD.
